# Errata

**Published:** 1892-01

**Authors:** 


**Errata.**—In our December number, the names of the writers were omitted from the two communications on "screw worms" in the nose. The letters were written, one by Dr. T. C. Osborn, Cleburne, and the other by Dr. T. J. Turpin, Laredo. The names appeared in the index only. We apologize to the gentlemen for this accident.

